# Functional impairments in ADHD in function of anxiety, depressive and somatic complains in Brazilian young adults

**DOI:** 10.1192/j.eurpsy.2021.1925

**Published:** 2021-08-13

**Authors:** L.R. Carreiro, M. Silva, I. Paes

**Affiliations:** Developmental Disorders Graduate Program, Mackenzie Presbyterian University, São Paulo, Brazil

**Keywords:** ADHD, Anxiety, Depression, Functional Losses

## Abstract

**Introduction:**

Comorbidities between Anxiety Disorders, Depressive Disorders or Somatic Symptoms, and Attention Deficit Hyperactivity Disorder (ADHD) can cause variability in the functional impairments faced by young adults. Knowing the possible configurations resulting from these comorbidities is important for a better understanding of the cases, diagnostic processes, and proposed treatments.

**Objectives:**

To verify associations between indicators of the aforementioned mental disorders, and symptoms of inattention or hyperactivity-impulsivity, and functional impairments in different areas of life, related to ADHD.

**Methods:**

There were 27 participants (23 women, age m = 22.5 sd = 1.8, education m = 15.7 sd = 2.2), with complaints of inattention and hyperactivity-impulsivity compatible with ADHD, screened with ASRS-18 score> 24 and WASI IQ> 79, and assessed by DIVA-2.0 (symptoms of ADHD), ASR-ASEBA (depressive, anxiety and somatic problems), EPF-ADHD (functional impairments in the academic, professional, affective, domestic, social, health, financial, traffic areas and legal risk). Spearman’s Correlation analysis was performed in the SPSS program (significance p <0.05).

**Results:**

Increase in depressive problems associated with increased symptoms of inattention (rho=0.386, p=0.049) and hyperactivity-impulsivity (rho = 0.406, p = 0.036). Increased somatic problems associated with increased functional impairment in health (rho=0.458, p=0.016). Increase in depressive problems associated with increased losses in the academic (rho=0.437, p=0.023), affective (rho=0.408, p=0.034), domestic (rho=0.550, p=0.002), social (rho=0.445, p=0.002), financial (rho=0.389, p=0.045) and health (rho=0.514, p=0.006).

**Conclusions:**

ADHD with comorbidities can have a peculiar clinical evolution with specific characteristics, including diagnosis, management, and response to treatment. These subgroups with different intervention needs demand outlining needs and personalized treatment.

**Disclosure:**

No significant relationships.

